# Helminth infection protects against high fat diet-induced obesity via induction of alternatively activated macrophages

**DOI:** 10.1038/s41598-018-22920-7

**Published:** 2018-03-15

**Authors:** Chien wen Su, Chih-Yu Chen, Yali Li, Shao Rong Long, William Massey, Deepak Vijaya Kumar, W. Allan Walker, Hai Ning Shi

**Affiliations:** 1 Mucosal Immunology and Biology Research Center, Massachusetts General Hospital and Harvard Medical School, Charlestown, USA; 2 Laboratory for Lipid Medicine and Technology, Department of Medicine, Massachusetts General Hospital and Harvard Medical School, Charlestown, USA; 3 Genetics and Aging Research Unit, Department of Neurology, Massachusetts General Hospital and Harvard Medical School, Charlestown, USA

## Abstract

Epidemiological studies indicate an inverse correlation between the prevalence of the so-called western diseases, such as obesity and metabolic syndrome, and the exposure to helminths. Obesity, a key risk factor for many chronic health problems, is rising globally and is accompanied by low-grade inflammation in adipose tissues. The precise mechanism by which helminths modulate metabolic syndrome and obesity is not fully understood. We infected high fat diet (HFD)-induced obese mice with the intestinal nematode parasite *Heligmosomoides polygyrus* and observed that helminth infection resulted in significantly attenuated obesity. Attenuated obesity corresponded with marked upregulation of uncoupling protein 1 (UCP1), a key protein involved in energy expenditure, in adipose tissue, suppression of glucose and triglyceride levels, and alteration in the expression of key genes involved in lipid metabolism. Moreover, the attenuated obesity in infected mice was associated with enhanced helminth-induced Th2/Treg responses and M2 macrophage polarization. Adoptive transfer of helminth-stimulated M2 cells to mice that were not infected with *H. polygyrus* resulted in a significant amelioration of HFD-induced obesity and increased adipose tissue browning. Thus, our results provide evidence that the helminth-dependent protection against obesity involves the induction of M2 macrophages.

## Introduction

The incidence of obesity is rising globally and remains one of the biggest threats to adults and children. More than one-third of U.S. adults suffer from obesity and 17% children were reported either overweight or obese in 2015^1^. Rising obesity rates have significant health consequences, contributing to increased rates of metabolic diseases such as diabetes, steatosis, hypertension, and heart disease. Obesity is characterized by chronic low-grade inflammation in adipose tissues (AT) from accumulating inflammatory cells (Th1/Th17 cells, macrophages, etc)^2^ which represents the unique feature of this metabolic inflammatory state. AT inflammation was proposed as a central mechanism connecting obesity with its metabolic and vascular complications. Various mediators and cytokines such as tumor necrosis factor alpha (TNF-α), interleukin-6 (IL-6), and monocyte chemoattractant protein-1 (MCP-1), released by the accumulating inflammatory cells and adipocytes, may contribute to the development of metabolic syndrome, thus exacerbating the consequences of obesity^3^.

In recent years, adipose tissue has emerged as a highly active body tissue integrating metabolic, endocrine, and immune functions into a single entity that exerts significant effects on whole body homeostasis. Under physiological conditions, the structural and functional integrity of AT are sustained by a meticulously orchestrated network of immune cells^4^. Resident immune cells constitute the second largest AT cellular component after adipocytes, and as such play important roles in the maintenance of AT homeostasis. Adipose tissue from lean individuals preferentially secretes anti-inflammatory adipokines such as adiponectin, transforming growth factor beta (TGFβ), IL-10, IL-4, IL-13, IL-1 receptor antagonist (IL-1Rα), and apelin. In contrast, obese adipose tissue mainly releases proinflammatory cytokines among other molecules such as TNF-α, IL-6, leptin, visfatin, resistin, angiotensin II, and plasminogen activator inhibitor 1^5^. The metabolic role of adipose tissue is complex. White adipose tissue (WAT) stores energy in the form of triglycerides. Brown adipose tissue (BAT) dissipates stored energy as heat, due to the presence of uncoupling protein 1 (UCP1), an integral membrane protein unique to brown adipocyte mitochondria^6^, which plays an important role in energy balance. BAT, therefore, increases energy expenditure and resistance to obesity. It has been shown that UCP1 transgenic mice, which have an increased content of UCP1, are protected against diet-induced obesity^7^.

Helminth infections form a major group of tropical diseases that affect approximately 1.5 billion people worldwide (or 24% of the world’s population)^8^. Concomitant to the decrease in parasitism over the past half century in developed countries, the prevalence of the so-called western diseases, such as allergic and autoimmune diseases, and metabolic syndrome have spectacularly risen^9^. This inverse correlation of immune mediated diseases and metabolic syndrome with helminth infections suggests that helminths may play a protective role against these diseases. Existing data indicate that helminth infections induce the development of Th2 and/or Treg responses, which have been linked to the amelioration of Th1- or Th17-mediated diseases^10,11^. The immune modulatory capacity of helminths has sparked a great deal of interest within the research community in exploring their therapeutic potential in treating immune-mediated diseases. Helminth-based therapy has been tested for controlling allergic and intestinal inflammatory disorders^2,12–16^. Despite these advances, our understanding of the potential mechanisms by which helminths modulate the host metabolism and nutrition-associated disorders remains incomplete.

Obesity is associated with macrophage accumulation in adipose tissue. The number of macrophages within adipose tissue differs depending on the metabolic status. Macrophages can exhibit either pro- or anti-inflammatory phenotypes and are routinely classified into classically activated M1 phenotype and alternatively activated M2 phenotype, respectively. Pro-inflammatory M1 macrophages secrete high levels of proinflammatory cytokines (TNF-α, IL-6, IL-1β) and generate reactive oxygen and nitrogen species, such as nitric oxide via activation of inducible nitric oxide synthase (iNOS). The alternatively activated M2 macrophages, which express Arg 1, Ym1/2, Fizz1 and produce IL-10, are anti-inflammatory^17–19^. Prior observations, including our own, have shown that helminth infection induces the development of alternatively activated macrophages (AAM or M2) via Th2 cytokines^17–19^. In the current study, we examine the effect of infection with the helminth parasite *Heligmosomoides polygyrus* on high fat diet (HFD)-induced obesity in mice and show that helminth-induced M2 macrophages play an important role in modulation of energy balance.

## Results

### *H. polygyrus* infection attenuated body weight gain, blood glucose and triglyceride levels in the HFD-fed obese mice

**Figure 1:**
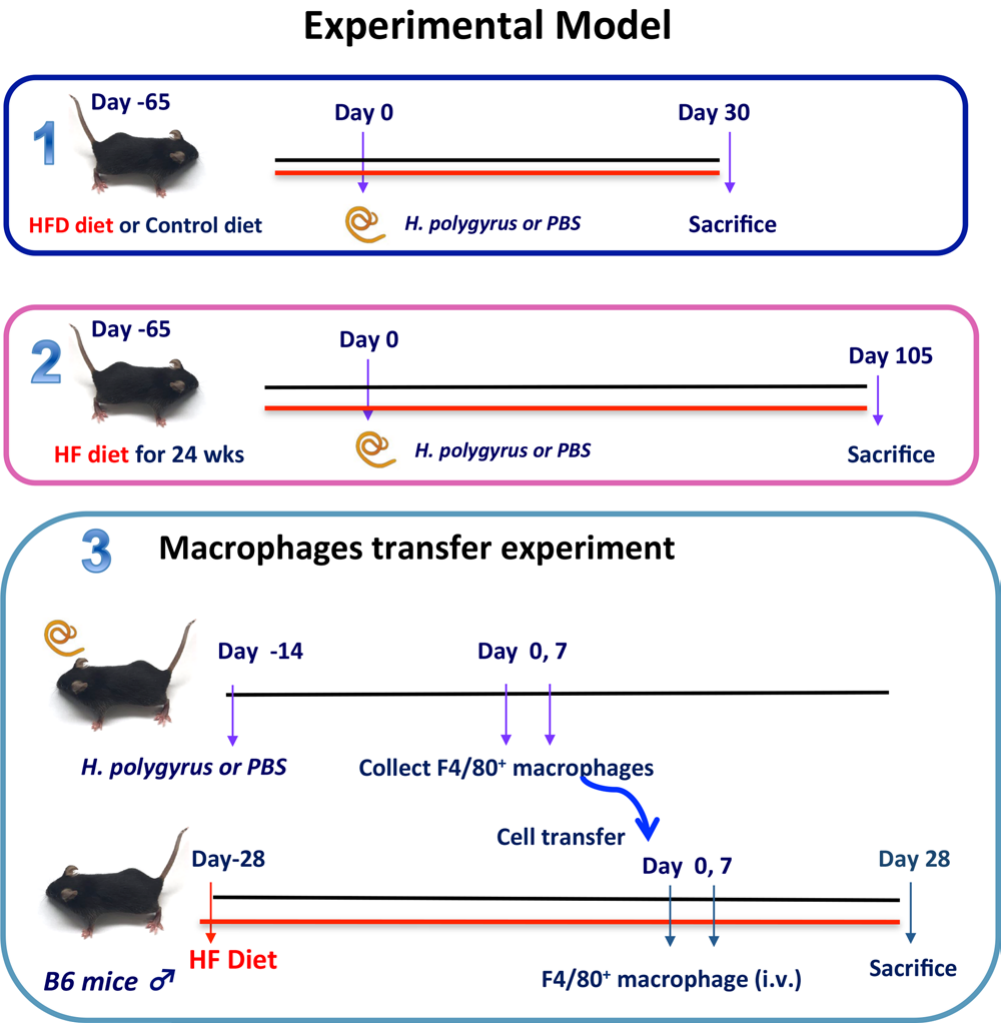
Experimental models. Schematic diagram illustrates *H. polygyrus* infection on HFD-fed mice. Schematic diagram illustrates *H. polygyrus* infection on long-term HFD experiment. The schematic diagram (3) illustrates the adoptive transfer of macrophage experiment in HFD-induced obesity; here the objective was to observe the potential regulatory role of helminth-induced M2 cells in HFD-obesity.

**Figure 2:**
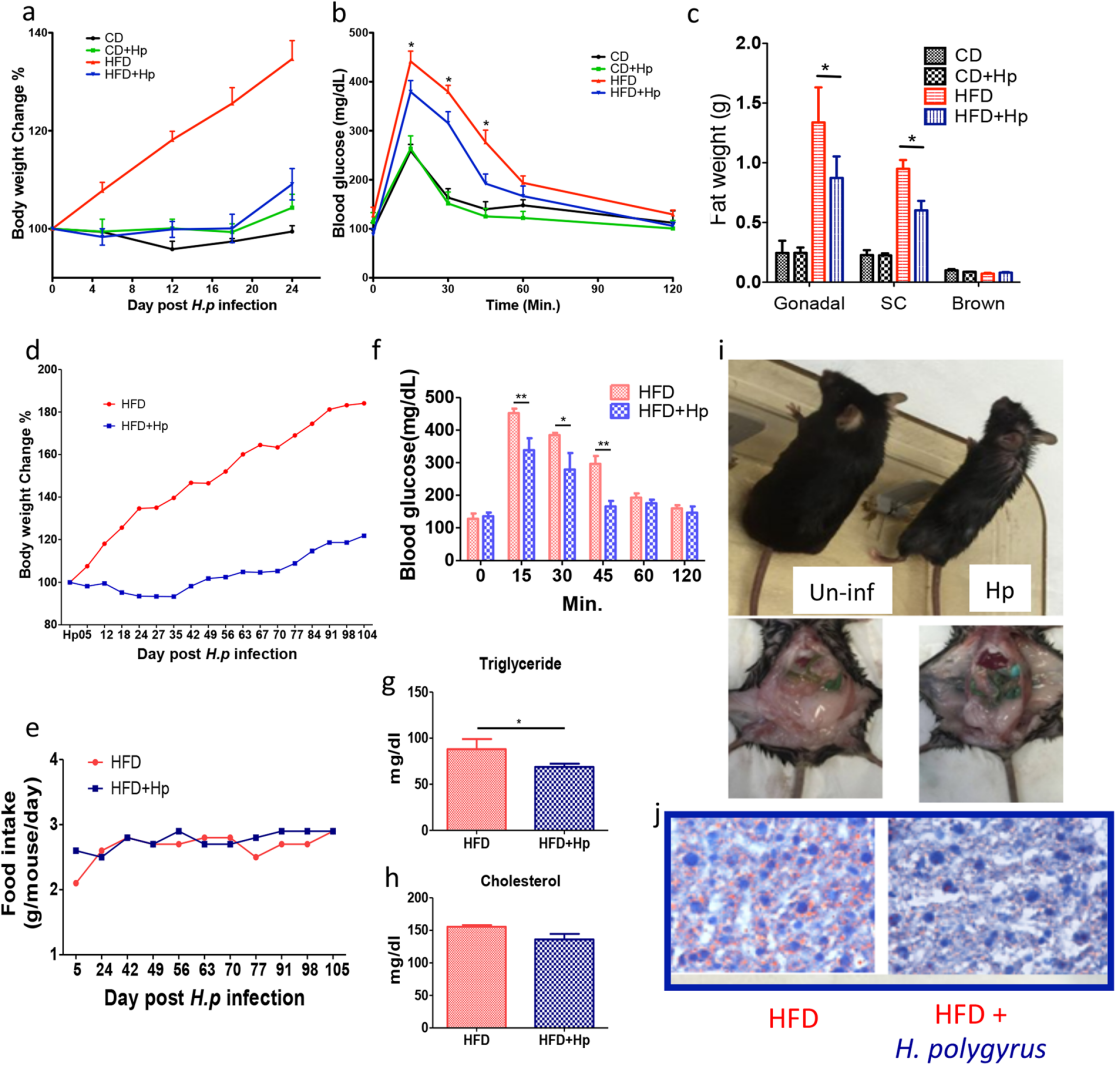
*H. polygyrus* infection attenuates HFD-induced obesity in mice. C57BL/6 mice fed with the HFD showed steady gain in body weight than those HFD infected with *H. polygyrus* through the course of experiment (**a**). Blood glucose levels were attenuated by *H. polygyrus* infection (**b**). Mice on the HFD accumulated more gonadal and subcutaneous fat than HFD fed infected mice (**c**). In long term experiments, C57BL/6 mice on HFD gradually gained more body weight (red line) than those HFD but infected with *H. polygyrus* (blue line) along a 104-day course (**d**). No differences in food intakes of HFD obese mice with (blue line) or without (red line) *H. polygyrus* infection (**e**). Blood glucose levels showed significant differences at 15, 30 and 45 minutes, between the two groups of mice (**f**). Triglyceride and cholesterol levels significantly dropped in *H. polygyrus* infected mice (**g** and **h**). The physical appearance (body size) in HFD-fed mice with (Hp) and without infection (Un-inf) (**i**) and the histological analysis of liver section of HFD induced mice showing severe steatosis, with fat deposits shown in un-infected mice using ORO staining (**j**). (n = 5–6; *p < 0.05; **p < 0.01. Two-tailed Student’s t-test).

To determine the effect of *H. polygyrus* infection on the HFD-induced obesity, C57BL/6 female mice were fed HFD or control diet followed by *H. polygyrus* or sham infection (Fig. 1). As expected, HFD feeding resulted in a significant increase in body weight gain in non-infected mice. In sharp contrast, as shown in Fig. 2a, *H*. *polygyrus*-infected, HFD fed mice gained significantly less weight compared to non-infected HFD fed mice. In fact, HFD-fed mice with helminth infection maintained their body weight at levels similar to mice fed with CD throughout the experimental period. *H. polygyrus* infection had no effect on body weight gain in CD fed mice.

Obesity is shown to exert detrimental effects on glucose homeostasis and lipid metabolism. To test this in our model, we next examined whether helminth-mediated attenuation of obesity reversed the detrimental effects associated with obesity. The results from glucose tolerance tests showed that when challenged with glucose, non-infected mice had a higher blood glucose level (Fig. 2b) and *H. polygyrus*-infected mice showed an enhanced glucose tolerance apparent at various time points of the tolerance test. Analysis of the body fat distribution was proportional to and in agreement with reduced body weight gain. Hp-infected mice had marked reduction in gonadal and subcutaneous fat pads: approximately 30% reduction was observed compared to non-infected HFD induced obese mice (Fig. 2c). However, no differences were detected in brown fat weight (Fig. 2c) between infected and non-infected mice fed with HFD.

To determine whether helminth infection alleviated obesity in mice on long-term HFD-feeding regime, we fed both helminth-infected and non-infected mice with HFD for over 104 days and observed that the infected mice gained significantly less body weight than the HFD-fed non-infected mice (Fig. 2d). However, the body weight changes were not correlated with a decrease in food intake since we found food intake did not differ significantly between both helminth-infected and non-infected mice on HFD (Fig. 2e). In the long-term experiments, we also found that helminth infection resulted in reduced blood glucose levels (at 15, 30 and 45 minutes) (Fig. 2f), decreased serum triglyceride levels (Fig. 2g) and unchanged cholesterol level (Fig. 2h) in HFD fed mice. Moreover, marked differences in physical appearance of mice (body size) were detected between *H. polygyrus*-infected and non-infected mice (Fig. 2i). The body size of HFD-fed mice was almost double the size of HFD-fed mice with *H. polygyrus* infection. Histological analysis of liver section of HFD induced mice revealed severe steatosis (Fig. 2j left panel). In contrast, less fat deposits were detected in *H. polygyrus*-infected obese group (Fig. 2j right panel). Our collective data suggested that helminth infection attenuates HFD-induced obesity in mice.

### *H. polygyrus* infection influences gene expression of key enzymes/mediators of lipid metabolism

**Figure 3:**
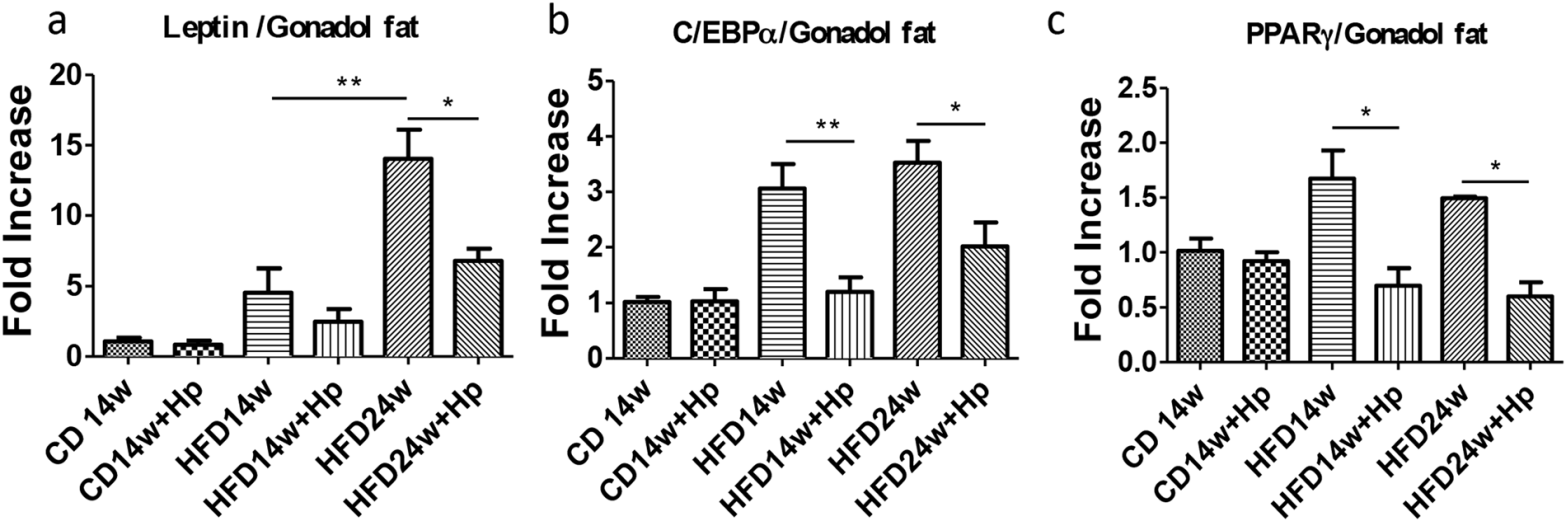
*H. polygyrus* infection influences gene expression of key enzymes/mediators of lipid metabolism. *H. polygyrus* infection results in decreased gene expression of leptin enzymes compared with normal mice in gonadal fat tissues: (**a**) leptin; (**b**) C/EBP*α*; and (**c**) PPARγ. (n = 5–6; *p < 0.05; **p < 0.01; two-tailed Student’s t-test).

To determine the mechanism by which helminth infection may alter HFD-induced body weight gain, we analyzed gene expression of key regulators mediating lipid metabolism in gonadal fat, including leptin, C/EBPα, and PPARγ. Leptin is a hormone produced in AT that regulates the amount of stored fat in the body by balancing both the appetite and energy usage^20^, and this adipocytokine has also been associated with pro-inflammatory attributes and the ability to induce pro-inflammatory cytokines such as IL-6, TNF-α, and IL-12^20^. PPARγ and C/EBPα are critical transcription factors in adipogenesis^21^. The results from our PCR analysis revealed that obese mice had higher leptin mRNA levels than the lean mice, and that HFD obese mice infected with *H. polygyrus* showed a marked decrease in leptin gene expression compared to that detected in non-infected HFD obese mice (Fig. 3a). Our results also showed that HFD-feeding resulted in significant increase in the expression of C/EBPα, which is prevented by helminth infection (Fig. 3b). The results suggest that helminth exposure in obese mice inhibited lipogenic gene expression. We further determined the impact of helminth infection on the expression of PPARγ and found that *H. polygyrus* infection significantly decreased the HFD-induced upregulation of PPARγ in obese mice (Fig. 3c). These results are in line with observations showing that PPARγ^−/−^ mice fail to generate adipose tissue when fed HFD^22^. Our observations suggest that *H. polygyrus* infection may ameliorate diet-induced obesity by modulating gene expression of key mediators of lipid metabolism

### *H. polygyrus* infection promotes the induction of UCP1 expression in both white adipose and brown adipose tissues

**Figure 4:**
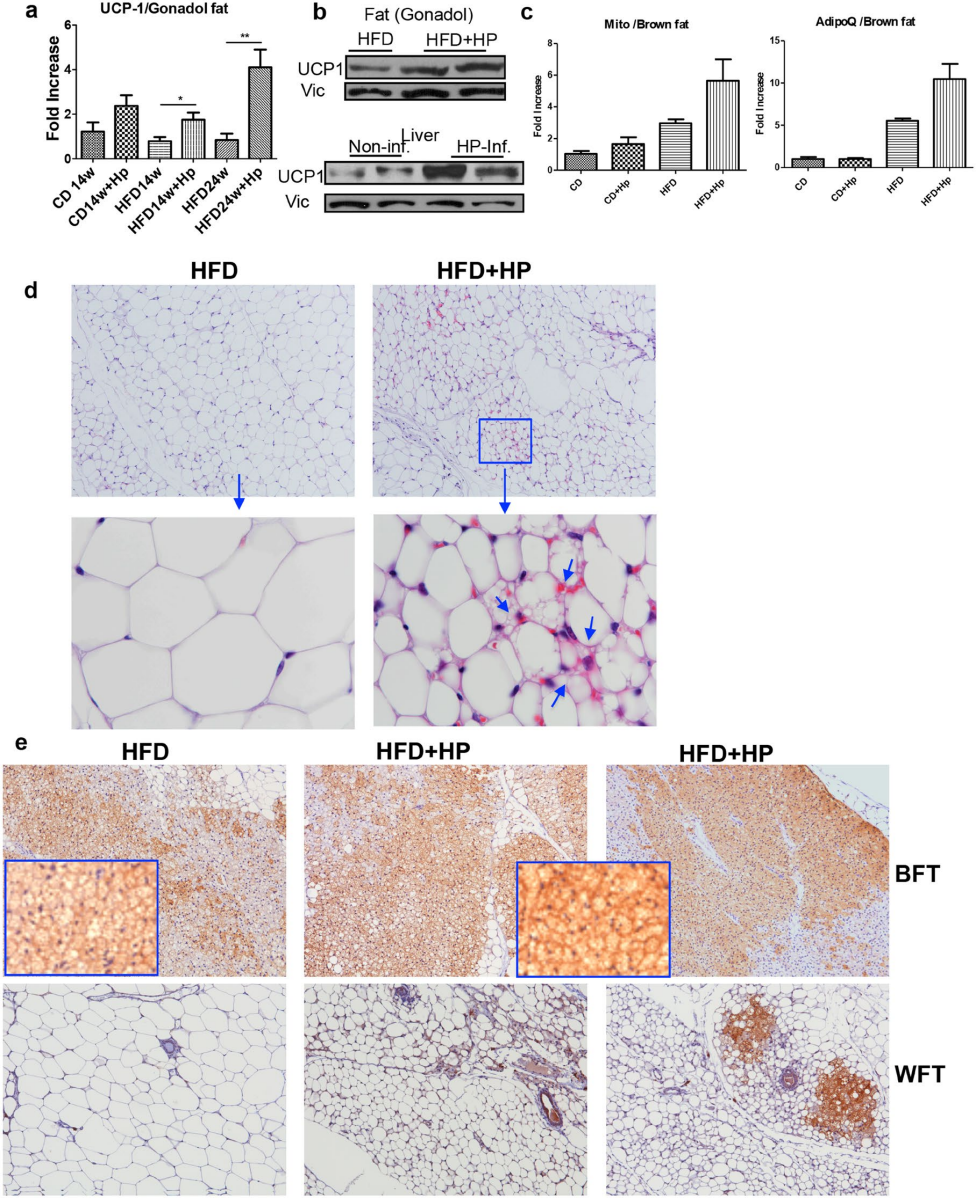
*H. polygyrus* infection results in upregulation of UCP1 expression and adipose tissue browning. qPCR analysis revealed that *H. polygyrus* infection results in a significant upregulation of UCP-1 expression in gonadal fat tissue at mRNA level (**a**). Immunoblot analysis showed increased UCP1 levels in adipose and liver tissue (**b**). qPCR results revealed significant upregulation of mitochondrial mass and UPC-1 expression (**c**). (n = 5–6; *p < 0.05; **p < 0.01; two-tailed Student’s t-test). (**d** and **e**) The adipose tissues showed marked reduction in the adipocyte size and increased expression of UCP1 in helminth-infected HFD fed mice (HFD-HP) compared to non-infected (HFD) mice. Adipose (including brown adipose, subcutaneous and gonadal adipose tissues) from HFD-fed mice with and without helminth infection collected. Ten-μm sections were cut on a Leica CM1850 Cryostat (Leica Biosystem) and were stained with hematoxylin and eosin (**d**) or anti-UCP1 antibody (**e**). (**d**) H&E staining: arrows indicate the presence of multilocular lipid droplets in adipocytes in the white adipose tissues of infected mice absent in uninfected obese mice. (**e**) Immunohistochemical staining of the brown (upper panel) and white (low panel) adipose tissues revealed a markedly intensified UCP1 expression in the adipose tissue of helminth-infected mice compared to non-infected HFD-fed mice.

Uncoupling protein 1 (UCP1), which is exclusively expressed in adipose tissue, particularly in BAT^23^, is involved in uncoupling- mediated energy expenditure and is a marker for fat browning. In our subsequent experiments, we tested the hypothesis that *H. polygyrus* infection promoted adipose tissue browning and contributed to increased resistance to diet-induced obesity. To test this idea, we first quantified UCP1 expression in different fat depots by using real time quantitative PCR (qPCR), immunoblot and immunohistochemical approaches. As illustrated in Fig. 4a,b, qPCR and immunoblot results revealed significant upregulation of UCP-1 expression at both mRNA and protein levels in adipose tissues of helminth-infected mice compared to HFD-fed control animals. Our PCR data also showed helminth infection resulted in increased mitochondrial mass, characteristic feature of beige fat^24^. Immunoblot analysis further demonstrated that *H. polygyrus* infection resulted in increased UCP-1 expression in liver tissue (Fig. 4b).

Obesity induces “whitening” of brown adipose tissue,which is characterized by larger lipid droplets, inflammation, and decreased mitochondrial respiration^25–27^. We next determined if helminth infection induced browning of white adipose tissues in mice. Our hematoxylin and eosin (H&E) staining of the adipose tissues showed marked reduction in the adipocyte size in helminth-infected HFD fed mice compared to non-infected mice (Fig. 4d). Furthermore, we observed the presence of multilocular adipocytes in the white adipose tissues of infected mice and the absence in non-infected obese mice. Such tissue remodeling - presence of multilocular adipocytes - are characteristics of beige adipose cells (Fig. 4d).

Using immunohistochemical approach, we determined the UCP-1 expression in white adipose tissue. Our analysis of the brown adipose tissue revealed that UCP1 expression was substantially intensified in the adipose tissue of helminth-infected mice compared to non-infected HFD-fed mice (Fig. 4e). These observations therefore supported that helminth infection enhances the UCP-1 expression in brown adipose tissues, which in turn may contribute to increased energy expenditure and attenuated diet-induced obesity in helminth infected mice. As illustrated in Fig. 4e (lower panel), consistent with the observations of multilocular adipocytes, we observed more pronounced UCP1 positive cells in white adipose tissues in helminth-infected mice compared to non-infected control mice (Fig. 4e). These observations, therefore, demonstrated that *H. polygyrus* infection induces UCP1 expression, promoting the browning of white adipose tissue in mice.

### *H. polygyrus* infection promotes a strong type 2 immune response resulting in cytokine-mediated modulation of lipid metabolism

**Figure 5:**
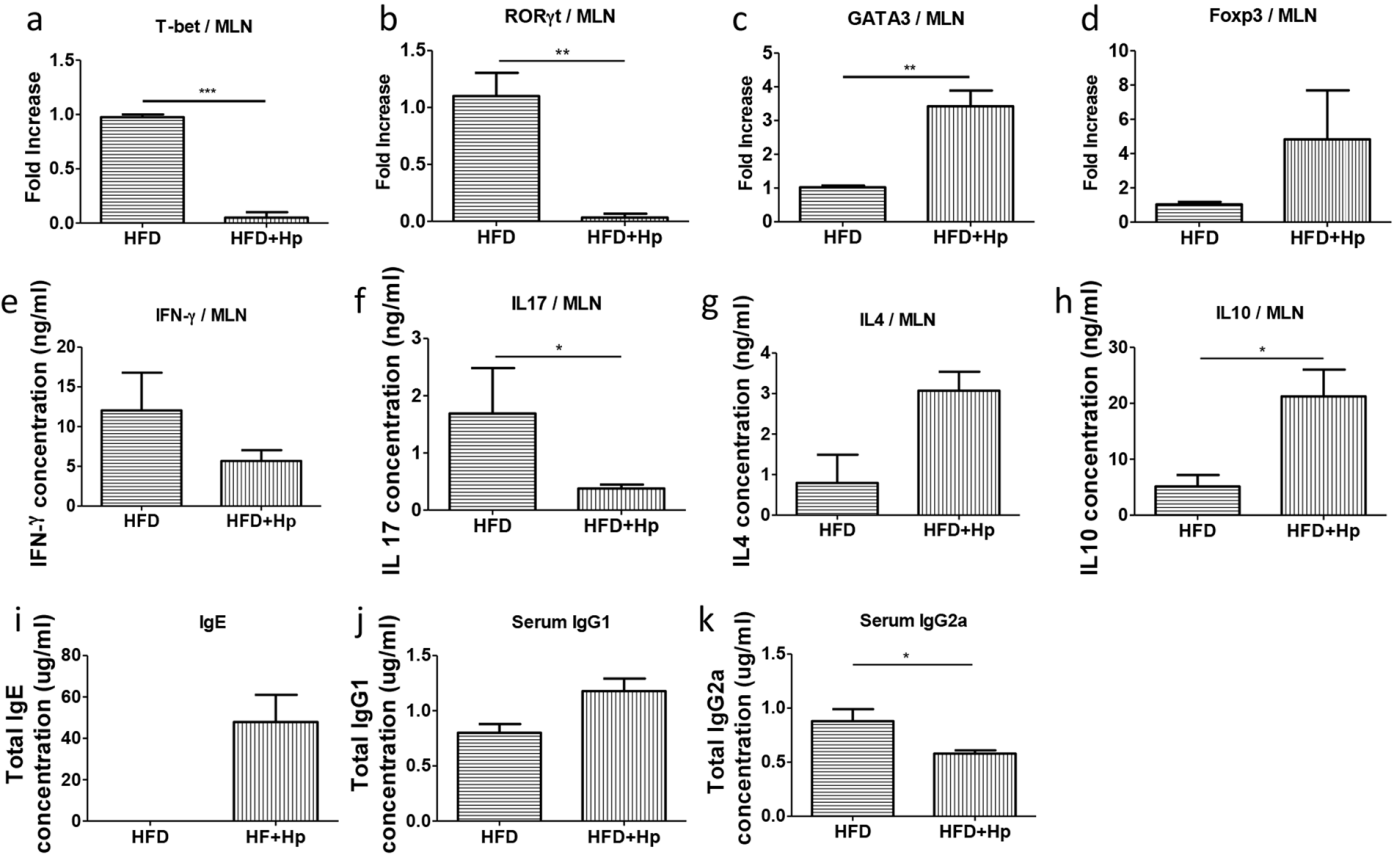
*H. polygyrus* infection promotes a strong type 2 immune response. Real time qPCR analysis shows a clear suppression in the T-bet and RORγt mRNAs expression (**a** and **b**) and increase in the expression of GATA3 and Foxp3 mRNAs (**c** and **d**) in the MLN cells from HFD-fed *H. polygyrus*-infected mice. *H. polygyrus*-infected obese mice showed decreases in IFN-γ and IL-17 secretions in the supernatant of cultured MLN cells (**e** and **f**). *H. polygyrus*-infected obese mice had higher production of cytokines IL-4 and IL-10 in MLN (**g** and **h**). *H. polygyrus*-infected obese mice had increased levels of serum IgE (**i**) and IgG1 (**j)** and a decreased IgG2a level (**k**). (n = 5–6; *p < 0.05; two-tailed Student’s t-test).

Cytokines TNFα, the IFNs, and IL-17A have been shown to regulate lipid metabolism^28,29^. In the liver, IFNs and other cytokines stimulate lipid synthesis and very-low-density lipoprotein (VLDL) production, which contribute to elevated serum triglyceride levels that characteristically occur during infections and inflammatory diseases. Evidence also showed that IL-17A is involved in lipid metabolism and pathogenesis of atherosclerosis, a chronic inflammatory arterial disease driven by both innate and adaptive immune responses to modified lipoproteins^30^. The helminth-induced immune responses mainly stem from the Th2 mediated cytokines IL-4, IL-10 and IL-13, the functions of which in nutrient metabolism and obesity are not fully understood and are an area of active research. In the current study, we collected MLN from different groups of mice and measured the expression levels of transcriptional factors responsible for the regulation and differentiation of Th1 (T-bet), Th2 (GATA3), Th17 (RORγt) and Treg (Foxp3) cells using real time qPCR. Our results show a clear suppression in the T-bet and RORγt mRNAs expression (Fig. 5a,b) and increase in the expression of GATA3 and Foxp3 mRNAs (Fig. 5c,d) in the MLN cells from HFD-fed *H. polygyrus*-infected mice. Furthermore, we re-stimulated the MLN cells *in vitro* and analyzed the cytokine profiles of the cells. Increased production of IL-4 and IL-10 were detected in the MLN cells from *H. polygyrus*-infected HFD-fed mice compared to non-infected obese host (Fig. 5g,h). The enhanced Th2 cytokine response in HFD-fed and helminth-infected mice corresponded with a marked reduction of IFN-γ and IL-17 production by MLN cells from these mice (Fig. 5e,f). In line with these observations, our further analysis of the impact of helminth infection on the immune system of HFD-fed mice revealed an enhanced systemic Th2 response and suppression of Th1 response, evidenced by increased serum IgE and IgG1 levels and decreased IgG2a levels (Fig. 5i,j,k). Taken together, our data showed a clear impact of on-going helminth infection on the effector T cell differentiation and cytokine production profile in mice fed HFD by promoting Th2/Treg and suppressing Th1/Th-17 cell responses.

### *H. polygyrus* infection enhances the ‘anti-inflammatory’ M2 levels by a balance shift in M1/M2 macrophage ratio

**Figure 6:**
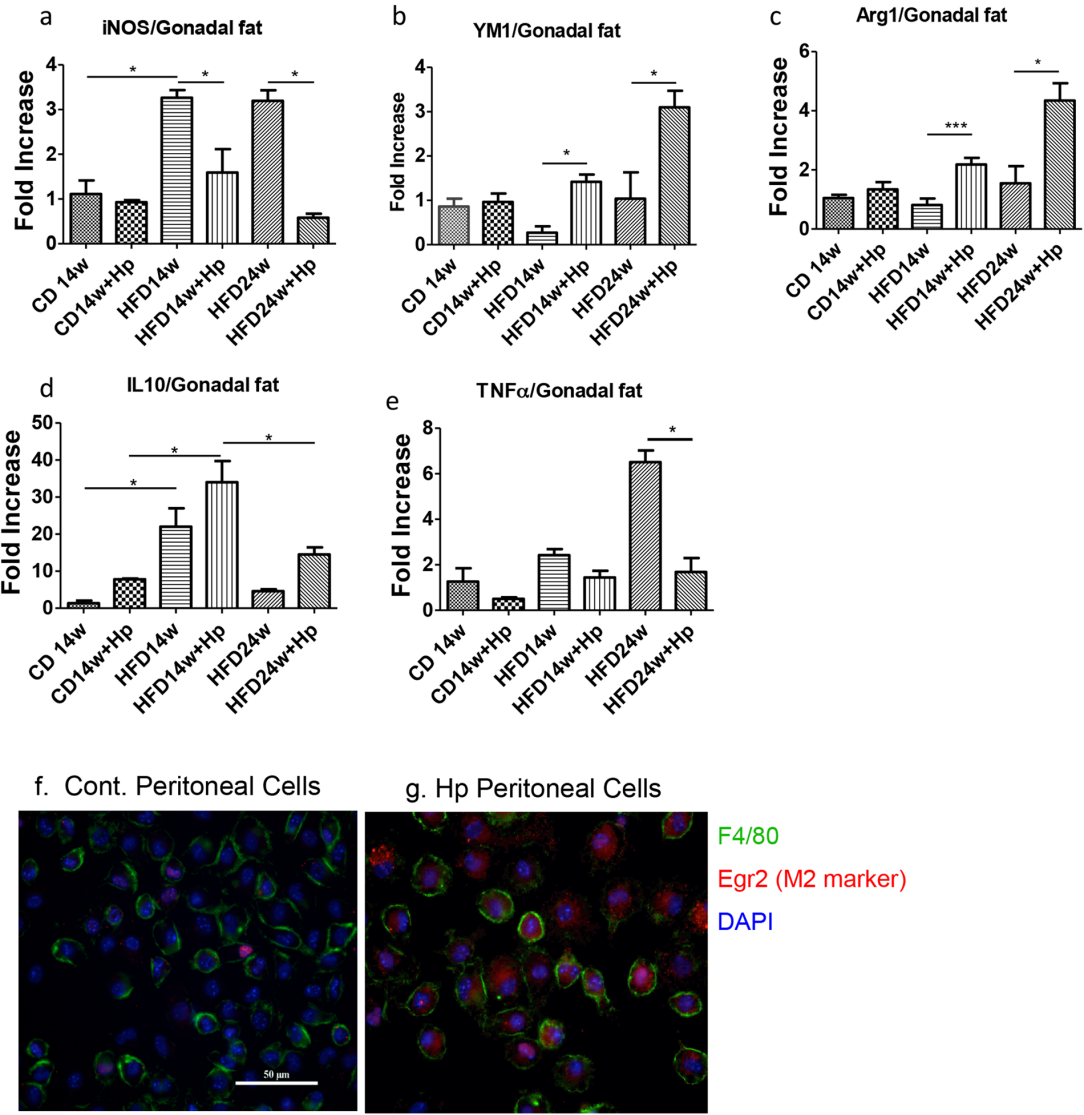
*H. polygyrus*-infection influences M1/M2 macrophage polarization in gonadal fat of obese mice towards anti-inflammatory M2 phenotype. (**a**) *H. polygyrus* infection results in reduced HFD-induced inducible oxide synthase (iNOS) expression, indicating inhibition M1 macrophages. (**b** and **c**) *H. polygyrus* infection results in increased expression of YM1 and Arg1, promoting the development of M2 cells in HFD-fed mice. (**d** and **e**) *H. polygyrus* infection results in increased IL-10 expression and decreased TNF-α expression. (n = 5–6; *p < 0.05; two-tailed Student’s t-test). (**f** and **g**) Peritoneal F4/80^+^ macrophages from normal control (**g**, mostly F4/80^+^EGR2^−^ M1) and *H. polygyrus*-infected mice (h: most F4/80^+^EGR2^+^ M2 cells).

Obesity is associated with macrophage accumulation in adipose tissue. The number of macrophages within adipose tissue differs depending on the metabolic status. In the current study, we determined whether helminth infection induces an imbalance in the ratio of M1/M2 macrophages in gonadal fat tissue. The results from our qPCR analysis showed that HFD-feeding of non-infected obese mice leads to increased M1 cells which are evidenced by significant increase in iNOS expression and this increase is prevented by helminth infection (Fig. 6a). The results from our qPCR experiments further indicated that *H. polygyrus* infection resulted in increased M2 macrophages in gonadal fat tissue as evidenced by the detection of enhanced expression of Arg1 and Ym1 levels, genes that are expressed in M2 cells (Fig. 6b,c). We also observed that *H. polygyrus*-infection significantly up-regulated IL10 expression in gonadal adipose tissues (Fig. 6e). The increased M2 and IL-10 responses corresponded with the decreased expression levels of the pro-inflammatory cytokine, TNF-α (Fig. 6e) and iNOS (Fig. 6a), suggesting a suppression of M1 cells in obese adipose tissue in helminth-infected HFD-fed mice. *H. polygyrus* infection induced M2 cells were further shown in peritoneal cell population from helminth-infected host, which is evidenced by the detection of high frequency of F4/80 and Egr2 double positive cells from infected host relative to control mice (Fig. 6f,g). These results suggest that *H. polygyrus* may protect against obesity by modulating the polarization of macrophages (promoting anti-inflammatory M2 macrophages) in gonadal fat of obese mice.

### Adoptive transfer of helminth-induced M2 cells results in attenuated HFD-induced obesity

**Figure 7:**
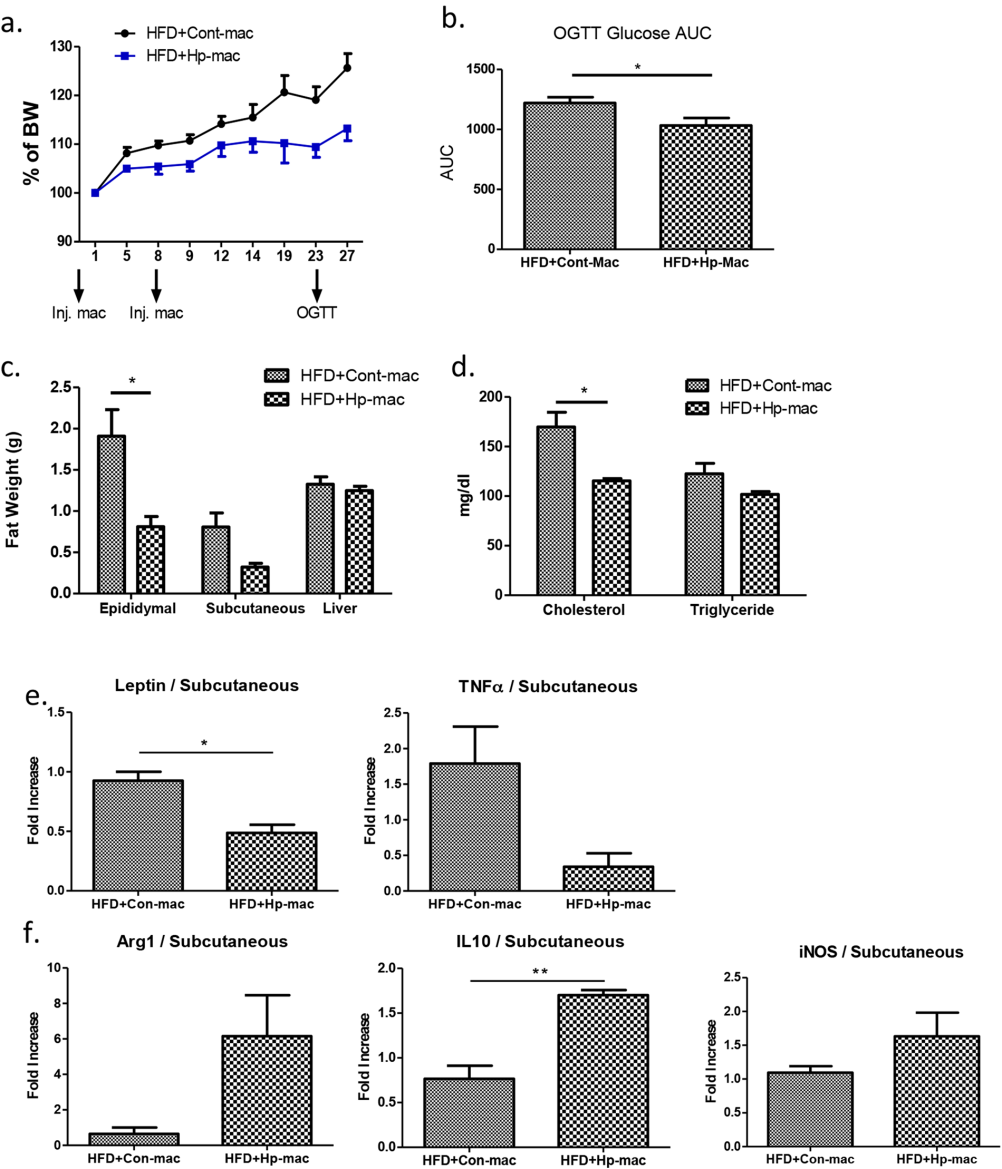
Adoptive transfer of M2 cells results in attenuated obesity in HFD-fed mice. (**a**) Body weigh change in mice received macrophages from normal and helminth-infected donor mice. (**b**) AUC of OGTT, Blood glucose level; (**c**) fat tissue and liver weight; (**d**) blood chemistry test; (**e**) M2 cell transfer alters gene expression of leptin and TNF-α; (**f**) M2 transfer displays M2 phenotype of increased IL-10 expression. (n = 5–6; *p < 0.05; **p < 0.01; two-tailed Student’s t-test).

To demonstrate the functional role of M2 cells in the regulation of HFD-induced obesity in our model, we isolated macrophages from helminth infected and normal control animals and adoptively transferred into the HFD-pre-fed obese mice and examined the body weight gain for 4 weeks. As shown in Fig. 1, both male and female mice were used in the experiments and developed similarly enhanced weight gain on the high fat diet. Both sexes were also equivalently protected from high fat-induced weight gain by helminth infection. However, since male mice gained weight more rapidly than females, we used only males in the macrophage transfer experiments in order to shorten their duration. HFD-fed mice that received macrophages from normal control mice showed sustained body weight gain after cell transfer **(**Fig. 7a). In contrast, HFD-fed mice that received macrophages from helminth infected mice gained significantly less weight during the experimental period **(**Fig. 7a). The observations demonstrate the functional role of helminth-induced M2 cells in the attenuation of HFD-induced obesity. In line with the reduced weight gain, adoptive transfer of macrophages from helminth-infected donors resulted in reduced glucose, fat, cholesterol level, leptin, and TNF-α levels (Fig. 7b–e). M2 cell transfer from helminth infected donor mice increased IL-10 expression in HFD-fed recipient mice (Fig. 7f). The results confirm that macrophages stimulated by helminth infection play an important role in modulating host metabolism, contributing to the amelioration of diet-induced obesity.

### Helminth-induced M2 cells promote UCP1 expression and browning of adipose tissues in mice

**Figure 8:**
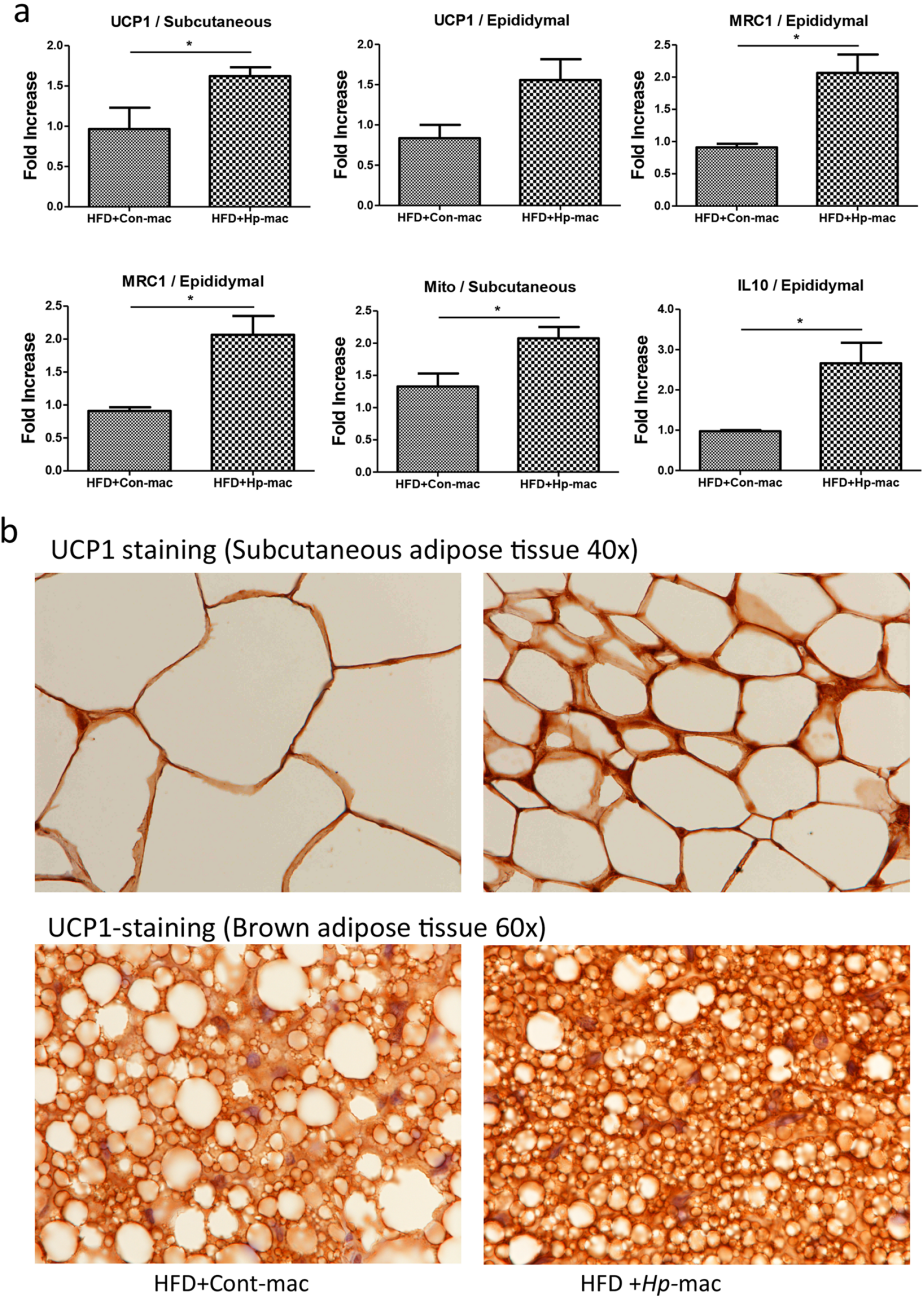
Adoptive transferring of *H. polygyrus-*induced M2 cells promotes adipose tissue browning. Purified F4/80^+^ macrophages from normal or *H. polygyrus*-infected donors were transferred into HFD-fed recipient mice. Adipose tissues were collected. (**a**) qPCR data showed significant increase in UCP1 expression and mitochondrial levels in adipose tissues from mice that received helminth-stimulated M2 cells. (n = 5–6; *p < 0.05; two-tailed Student’s t-test). (**b**) Representative of immunohistochemical stained sections showed marked reduction of the adipocyte size and increased intensity of UCP1 expression in the adipose tissue of the recipient mice transferred with helminth-induced M2 cells compared to mice harboring normal macrophages.

To test if *H. polygyrus-*induced M2 cells may promote adipose tissue browning, and contribute to increased resistance to dietary obesity, we examined the UCP1 expression and mitochondrial level in the recipient mice that were transferred with macrophages from normal or helminth infected donors. qPCR data confirmed a significant increase in UCP1 expression and mitochondrial levels in adipose tissues from mice that received helminth-stimulated M2 cells (Fig. 8a). qPCR analysis of the adipose tissue also confirmed that the recipient mice of macrophages from helminth infected donor showed M2 characteristics, as evidenced by increased MRC1 (CD206) and IL-10 expression (Fig. 8a), both of which are known to be expressed in M2 cells. Our immunohistochemical analysis of the adipose tissues showed marked reduction of the adipocyte size in mice that were subject to helminth-induced M2 cell transfer compared to mice that received cells from non-infected donor mice (Fig. 8b). Our results further showed that adoptive transfer of helminth-induced M2 cells increased UCP1 expression in the adipose tissue compared to mice harboring normal macrophages (Fig. 8b). The increased UCP-1 expression may contribute to enhanced thermogenesis. Overall the collective observations demonstrate a significant role for helminth-induced M2 cells in enhancing energy expenditure and attenuating dietary induced obesity in mice. These observations, therefore, suggest a new aspect of immune regulatory effects of helminth infection and provide mechanistic insights into our observation that *H. polygyrus* attenuate HFD-induced obesity.

## Discussion

Helminth infections form a major group of tropical diseases that affect approximately 1.5 billion people worldwide (or 24% of the world’s population)^8^. Success in eradicating helminth infection in the developed world significantly reduced helminth-associated morbidity and mortality, but is also correlated with increasing incidence of “western diseases”, including metabolic syndrome and type 2 diabetes^31^. This inverse correlation between the incidence of immune mediated diseases and metabolic syndrome and helminth infections suggests that helminths may play a protective role against these diseases. Our current findings show that infection with the small intestinal Th2-stimulating helminth parasite *H. polygyrus* ameliorates HFD-induced obesity. The amelioration of obesity in infected mice was accompanied by an intensified UCP1 expression, and increased browning of white adipose tissue, and altered gene expression of key regulators mediating lipid metabolism in gonadal fat. The results further showed that attenuated obesity in *H. polygyrus*-infected mice was associated with enhanced M2 macrophage polarization. By using adoptive cell transfer approach, we showed that transplantation of *H. polygyrus*-stimulated M2 macrophages to the HFD-fed recipient mice attenuated HFD-induced obesity, corresponding with enhanced UCP-1 expression in adipose tissues and an increase in number of beige cells (or browning) in adipose tissue. The results, therefore, demonstrate a functional role for helminth-induced M2 cells in orchestrating the host response to diet.

The mechanism of the relationship between helminth infection and nutrition is complex and remains to be fully elucidated. We show that a Th2-inducing intestinal helminth (*H. polygyrus*) infection has a beneficial effect on diet-induced obesity. It has been shown that injection of mice on a HFD with the Th2 cytokine IL-4 activates STAT 6 signaling pathway and attenuates adipose tissue inflammation, and improves insulin action^32^. IL-4 has also been suggested to play a pro-lipolytic role in lipid metabolism by enhancing the activity and translocation of hormone sensitive lipase (HSL) in mature adipocytes and by IL4-mediated inhibition of adipocyte differentiation and lipid accumulation, thereby promoting lipolysis in mature adipocytes (decreased lipid droplets)^32^. Eosinophils, which are associated with helminth infection, reportedly enhance glucose tolerance^33^. We have also observed helminth-infected HFD-fed mice have markedly elevated serum IgE levels, which have been associated with decreased fasting blood glucose and lipid levels^34^. Our results are consistent with prior reports that Th2-inducing nematode parasite *Nippostrongylus brasiliensis*^35^ and *Schistosoma mansoni*^36^ infection in HFD-fed mice resulted in decreased body weight gain and improved glucose metabolism. These results further support the inverse association between helminth infections and the incidence of metabolic syndrome^37^.

Diet-induced obesity can lead to a shift in the activation state of adipose tissue macrophages from an M2-polarized state in lean animals to an M1 proinflammatory state that contributes to insulin resistance^38^. This imbalance culminates in chronic inflammation and long-term metabolic dysfunction. The results from the current study showed that in HFD-fed mice *H. polygyrus* infection influences M1/M2 macrophage polarization in gonadal fat, shifting it towards a predominantly anti-inflammatory state or M2 phenotype. In line with our observation, M2 phenotype has also been detected in adipose tissue in mice with *N. brasiliensis* infection^35^. The detection of an M2-polorized state in helminth-infected HFD-fed lean mice may suggest that macrophages in helminth-infected mice migrating to adipose tissue upon high-fat feeding may contribute to altered lipid metabolism. To test this hypothesis, in the current study, we carried out the macrophage transfer experiments by collecting F4/80+ macrophages from helminth-infected or uninfected control donors and adoptively transferred the cells to HFD-fed recipient mice. We showed that adoptive transfer of helminth-stimulated M2 cells to mice that were not infected with *H. polygyrus* resulted in a significant amelioration of HFD-induced obesity and increased adipose tissue browning, demonstrating the functional contribution of helminth-induced M2 cells in the attenuation of HFD-induced obesity.

Our results showed that the food intake was not affected by helminth infection (Fig. 2e), suggesting the attenuated obesity in HFD-fed mice is independent of any pathology associated with helminth infection. Our results provide evidence that helminth infection results in marked upregulation of UCP-1 and mitochondrial expression, activated brown adipose tissue and increased browning of the white adipose tissues in HFD-fed mice, which may contribute to increased energy expenditure, consequently, limited adiposity (Fig. 4). These observations, therefore, provide new mechanistic insights into *H. polygyrus* induced attenuation of HFD-induced obesity. UCP1 allows brown adipose tissue to generate heat via uncoupling oxidative phosphorylation at the expense of ATP production. New evidence suggests that post transcriptionally stabilizing UCP1 mRNA protects mice from HFD-induced obesity^38^. Loss of UCP1 resulted in whitening of brown adipose tissue and exacerbates Western diet-induced glucose intolerance^39^. Brown adipose tissue has been associated with protection against inflammation and inversely associated with insulin resistance, Type 2 diabetes, and risk of cardiovascular disease^40^. In line with our observation that helminth infection stimulates the browning of white adipose tissues, this process has been shown to occur under certain environmental and pharmacological conditions. For example, cold exposure stimulates the browning of white adipose tissues^41,42^. Increased UCP1 activity and mitochondrial biogenesis in white AT have also been shown as results of exercise training^43,44^. During the exercise training, it was shown that β-adrenergic activation^43,44^ improved insulin sensitivity and increased energy expenditure^45,46^. Helminth infection may activate adipocyte precursors to adopt a beige fate through induction of the cytokines IL-4/IL-13 and ILC2s^9^, as these Th2 promoting molecules and cells have been implicated in the commitment and maturation of beige adipocytes^47,48^. In contrast, there is evidence from a recent study showing that loss of ILC2 and Treg did not affect uncoupled respiration in brown or beige adipocytes^49^.

Similar to our observations showing that helminth infection results in a marked upregulation of UCP-1 expression, the results from our macrophage transfer experiments provide strong evidence that transfer of helminth-induced M2 cells, in the absence of helminths in the recipient host, is sufficient to induce an attenuated obesity and enhanced UCP-1 expression in adipose tissues, leading to the activation of browning in adipose tissue and the potential for enhanced thermogenesis. These novel observations suggest that the observed helminth-induced protective metabolic effects involve browning of adipose tissue by helminth-stimulated M2 cells. Previous study has demonstrated that cold exposure promotes M2 cell development^50^. M2 cells have been implicated in activation of beige adipocytes to generate heat by producing catecholamines^50^. The question how precisely helminth-stimulated M2 regulates the UPC1 expression warranties a future investigation.

In our current study, an increased expression of the prototypical anti-inflammatory cytokine, IL-10, was observed in adipose tissues of helminth-infected obese mice. The same is true in M2 cell recipient mice, which may suggest that helminth-stimulated M2 cell derived IL-10 constitutes a major portion of the IL-10 in adipose tissue. Overexpression of IL-10 in macrophages suppresses atherosclerosis in hyperlipidemic mice^51^. IL10 has been shown to be able to ameliorate inflammation in obese adipose tissue and insulin resistance induced by proinflammatory cytokines, TNF-α and MCP-1^52,53^. IL-10 can modulate lipid metabolism enhancing both uptake and efflux of cholesterol in macrophages, playing an antiatherogenic role^54^.

Our histological analysis of liver section of HFD induced mice revealed severe steatosis. However, much less fat deposits were detected in *H. polygyrus*-infected obese group (Fig. 2i). Although the precise mechanism by which helminth infection regulates liver disease is still unclear, we speculate that the marked attenuation of liver damage may be related to helminth-induced Th2/Treg cell homing to the lever, dampening Th1/Th17 inflammatory response. It has been suggested that mucosal DC-primed T cells can home to liver, and T cells activated by liver sinusoidal endothelial cells are capable to home to the intestine, termed as “two-way cross talk” in the gut-liver axis^55^. As over 80% the body’s macrophages reside in the liver^56^, it is highly likely that helminth-induced AAM may aid in the repair of damage liver tissues^56^.

The significant inhibitory effect of *H. polygyrus* infection on diet-induced obesity in our model supports the idea that helminth parasites, which infect millions of people worldwide, particularly in the developing world, may have beneficial metabolic effects. Our results also support the potential for helminths as a new class of biologics in treating inflammatory diseases and metabolic disorders. Further identification of the specific immunomodulatory components of helminths may catapult it to a more refined treatment strategy against obesity and other inflammatory diseases. Our recent published work showed that *H. polygyrus* infection induces significant alterations in intestinal microbiota composition and functionality^57^. However, the potential role for helminth-altered microbiota in the protection against HFD-induced obesity remains to be determined. Taken together, results from our study raise the intriguing possibility of using helminths as novel, safer and effective therapeutics in the treatment of obesity and other immune and metabolic disorders.

## Methods and Materials

### Mice and diet

Four-week-old female C57BL/6 mice were purchased from The Jackson Laboratory (Bar Harbor, ME), fed a normal control diet (CD, 10% of kcal from fat) or given a high-fat diet (HFD, 60% of kcal from fat; Research Diets, New Brunswick, NJ) to induce obesity. These mice were maintained in a specific-pathogen-free (including helminths such as pinworm) facility at Massachusetts General Hospital. Animal care was provided in accordance with protocols approved by the Institutional Animal Care and Use Committee of Massachusetts General Hospital. At different time points after dietary treatment was started, some of the HFD- or CD-fed mice were infected with *H. polygyrus*. The dietary treatments were maintained throughout the experimental period (Fig. 1). The body weight changes were measured throughout the experimental period and represented as a percentage of weight gain by dividing each mouse’s initial weight.

### *H. polygyrus* infection

*H. polygyrus* were propagated as previously described and stored at 4 °C until use^58^. Mice were inoculated orally with 200 third stage larvae.

### Glucose and insulin tolerance

The mice were fasted overnight for 15 hrs and challenged with glucose solution (2 g/kg of body weight) by oral gavage. Prior to glucose load, blood glucose level was measured and then monitored at times 15, 30, 45, 60, and 120 min, using a Bayer contour (Bayer healthcare LLC, Mishawaka, IN).

### Adiposity-specific gene expression

Total RNA was isolated from gonadal adipose tissue. Real time PCR (RT-PCR) was used to determine the expression levels of adipocyte specific genes (peroxisome proliferator-activated receptor gamma (PPARγ), leptin, cytokine genes (IL-6, IL-10 and TNF-α), M1 macrophage marker (iNOS2) and M2 macrophage markers (CD206, arginase1, Ym1). mRNA expression of markers of adipocyte differentiation (CCAAT/enhancer-binding protein alpha, C/EBPα) was measured in visceral fat depots (mesenteric fat) and brown fat. GAPDH was the housekeeping control.

### Measurement of cytokine production

Th1 (IFN-γ), Th2 (IL-4), Treg (IL-10) and Th17 (IL-17) cytokines were assayed using ELISA as previously described^59^. ELISA capture (BVD4-1D11, IL-4; R4-6A2, IFN-γ; IL-10; IL-17) and biotinylated second Abs (BVD6-24G2, IL-4; XMG1.2, IFN-γ; IL-10; IL-17) was purchased from BD Pharmingen. Standard curves were obtained using recombinant murine IFN-γ; IL-4; IL-10; and IL-17 (Genzyme, Cambridge, MA, US).

### Measurement of serum antibody

Each mouse was bled at sacrifice, and individual sera were assayed for total IgE, IgG1 and IgG2a by ELISA on plates coated with goat anti-mouse Ab to IgG1, IgG2a (BD PharMingen) or rat anti-mouse Ab to IgE (BD PharMingen). Blocked, washed plates were incubated with diluted serum samples in triplicate for 1.5 h at RT. After washing with PBS/Tween, IgG1 and IgG2a were detected with HRP-conjugated goat anti-mouse IgG1, IgG2a, respectively (PharMingen); IgE was detected with biotin-conjugated rat anti-mouse IgE (PharMingen) and peroxidase-conjugated streptavidin (Zymed Labs.). Both reactions were developed with OPD and read at 492 nm. OD values were converted to μg/ml of IgG1, IgG2a and IgE by comparison with standard curves of purified IgG1, IgG2a (PharMingen) or IgE (PharMingen) by linear regression analysis, and are expressed as the mean concentration for each group of mice ± SEM.

### Histopathological examinations

At necropsy, liver and fat tissues from helminth-infected, non-infected were collected, frozen in Tissue Tek OCT compound (Miles, Inc., Elkhart, IN) and stored at −80 °C. Ten-μm sections were cut on a Leica CM1850 Cryostat (Leica Biosystem) and were stained with hematoxylin and eosin or anti-UCP1 antibody. For the fat tissues, the samples from brown adipose, subcutaneous and gonadal adipose tissues were prepared. Using the histopathological approach, we examined the sizes of adipocytes from the various groups of mice that were fed with HFD or CD with and without *H. polygyrus* infection. Both brown and white adipose tissue sections were processed and UCP1 expression of adipose tissues was determined using immunohistochemical staining with anti-UCP1 antibody (ab10983, Abcam, Cambridge, MA, US).

### Macrophage isolation and adoptive transfer

Spleen and peritoneal cells from *H. polygyrus*-infected mice (2–3 weeks post infection) and non-infected mice were collected aseptically into complete DMEM as previously described^59^. The tissues were digested with collagenase (type IV; Worthington Biochemical) at 200 U/ml for 45 min at 37 °C. Macrophages were purified by positive selection over a magnetic cell-sorting column (MACS) using microbead-conjugated anti-F4/80 mAb (Miltenyi Biotec). Purified F4/80^+^ macrophages from normal or *H. polygyrus* infected donors were transferred into HFD-fed recipient mice (4 weeks on the dietary treatment) (2–3 × 10^6^ cells per mouse) via tail vein injection. HFD treatment was sustained for 4 weeks after macrophage transplantation. Immunostaining with anti-F4/80 and anti- early growth response factor-2 (EGR2) was used to confirm the M2 phenotype of macrophages from helminth-infected mice.

### Statistical analysis

All results were expressed as the mean ± standard error of the mean. N refers to the number of mice used. Statistical differences were determined using a two-tailed Student’s t-test (GraphPad Software, Inc., San Diego CA.). A *p* value of <0.05 was considered significant.

### Data availability

All data generated or analysed during this study are included in this article.
